# Treatment of Necrotic Calcified Tooth Using Intentional Replantation Procedure

**DOI:** 10.1155/2014/793892

**Published:** 2014-03-04

**Authors:** Nima Moradi Majd, Armita Arvin, Alireza Darvish, Sareh Aflaki, Hamed Homayouni

**Affiliations:** ^1^Department of Endodontics, Dental School, Qazvin University of Medical Sciences, Qazvin 34157-59811, Iran; ^2^Department of Endodontics, Dental School, Yazd University of Medical Sciences, Yazd 8914881167, Iran

## Abstract

*Introduction*. If the teeth are impacted by a chronic irritant, the pulp space possibly will undergo calcific changes that may impede access opening during root canal treatment. In such cases that conventional endodontic treatment is impossible or impractical, intentional replantation may be considered as a last solution to preserve the tooth. *Methods*. After failing to perform conventional root canal therapy for a necrotic calcified right mandibular second premolar, the tooth was gently extracted. The root apex was resected and the root end cavity was prepared and filled with calcium enriched mixture (CEM) cement. Then, the extracted tooth was replanted in its original position. *Results*. After a year the tooth was asymptomatic, and the size of periapical radiolucency was remarkably reduced and no clinical sign of ankylosis was observed. *Conclusion*. Intentional replantation of the necrotic calcified teeth could be considered as an alternative to teeth extraction, especially for the single-rooted teeth and when nonsurgical and surgical endodontic procedures seem impossible.

## 1. Introduction

The root canal systems of the teeth usually remain patent and accessible, but if they are impacted by a chronic irritant, the pulp space possibly will undergo calcific changes that may impede access opening during root canal treatment [1].

Although pulp space of this kind of teeth sounds completely obliterated in preoperative radiographs, this space has adequate room to allow passage of millions of microorganisms [2]. Therefore, a calcified tooth with pulp necrosis inevitably leads to induction of apical periodontitis [1].

The first option for treatment of a calcified necrotic tooth is the conventional root canal therapy [3], but teeth with severe calcification may present challenges with locating and negotiating root canals. The other options beside nonsurgical endodontic treatment include root resection using a surgical method [4] and intentional extraction and replantation [5].

Intentional replantation procedure is usually considered as a last resort [6], but in some cases that conventional endodontic treatment or apical surgery is impossible or impractical, intentional replantation may be considered as a solution to preserve the tooth [6].

The present case report describes a successful treatment of a calcified necrotic mandibular second premolar using intentional replantation procedure.

## 2. Case Presentation

**Figure 1:**
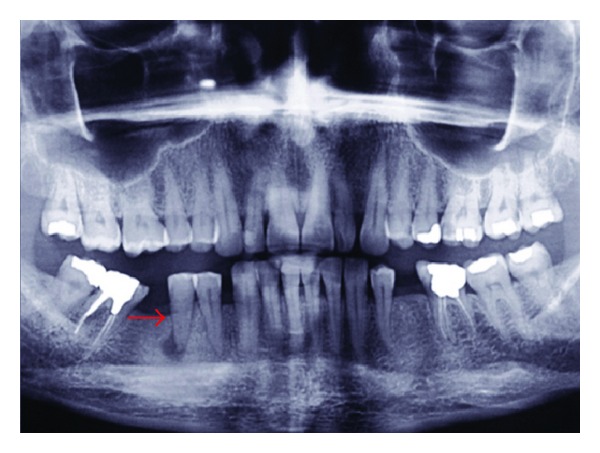
Preoperative radiograph; the pulp space of the right second mandibular premolar has been seriously obliterated and periapical radiolucency is observed at the apex of the tooth.

A 44-year-old female with no contributing medical history was referred to the Endodontic Department of Qazvin school of Dentistry. She stated that her right mandibular second premolar hurt when she chews. After clinical examination, moderate tooth attrition on the occlusal surface of the tooth was observed. The tooth was moderately sensitive to percussion, but neither sinus tract nor periodontal pocket was detected. Radiographic examination revealed that the pulp space has been seriously obliterated. In addition, periapical radiolucency was observed at the apex of the right mandibular second premolar (Figure 1).

The tooth was examined by electric pulp test (EPT) using the Element Diagnostic Unit (SybronEndo, Glendora, CA) and cold test (Roeko Endo-Frost; Roeko, Langenau, Germany).

The presence of the periapical radiolucency and tooth's negative responses to EPT and cold test convinced us that the right mandibular second premolar is a necrotic tooth that needs to undergo conventional root canal therapy.

**Figure 2:**
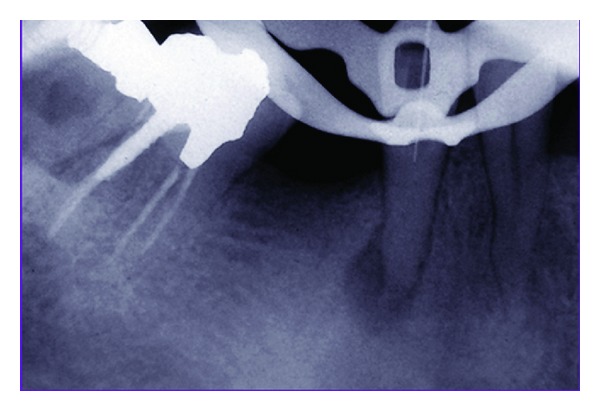
In order to find root canal's orifice, some working radiographs were taken, but root canal negotiation was impossible.

**Figure 3:**
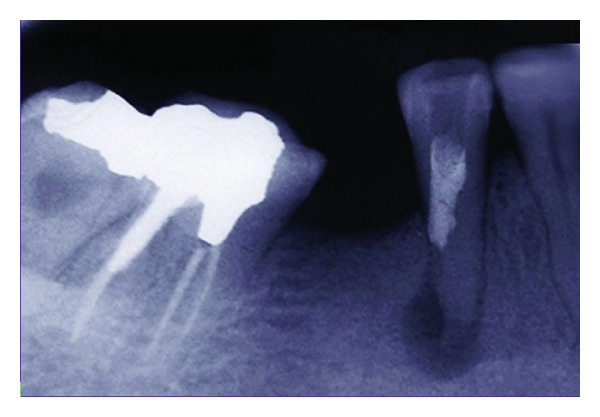
Calcium enriched mixture cement was used to seal the perforation.

The inferior alveolar nerve block (IANB) was carried out using a cartridge of lidocaine (2% lidocaine with 1/80000 epinephrine; Darupakhsh, Tehran, Iran); after proper isolation, an endodontic postdoctoral student attempted to prepare an endodontic access at lower right second premolar with a round bur, but at the normal anatomical orifice level, no sign of orifice was found. He continued trying to find the root canal's opening (Figure 2), but when he was attempting to negotiate the calcified canal, a perforation was created on the distal root surface 1 mm below the alveolar crest. The perforation was sealed using calcium enriched mixture (CEM) cement (BioniqueDent, Tehran, Iran) (Figure 3), and the access cavity was sealed with Cavit (coltosol, AriaDent, Tehran, Iran). The next treatment's options (apicoectomy, intentional replantation, extraction, and implant replacement) and their risks and benefits were described to the patient; we explained that apicoectomy is more predictable than intentional replantation, but there is danger of damaging contents of mental foramen; but the patient was going to consult her dentist about the treatment plan.

On the next day, the patient called the endodontic department and told us she consulted with her dentist and decided to do the intentional replantation. Thus, we arranged an appointment and restored the access cavity of the tooth number 29 using composite restoration. A written informed consent was obtained and she was scheduled for the intentional replantation.

**Figure 4:**
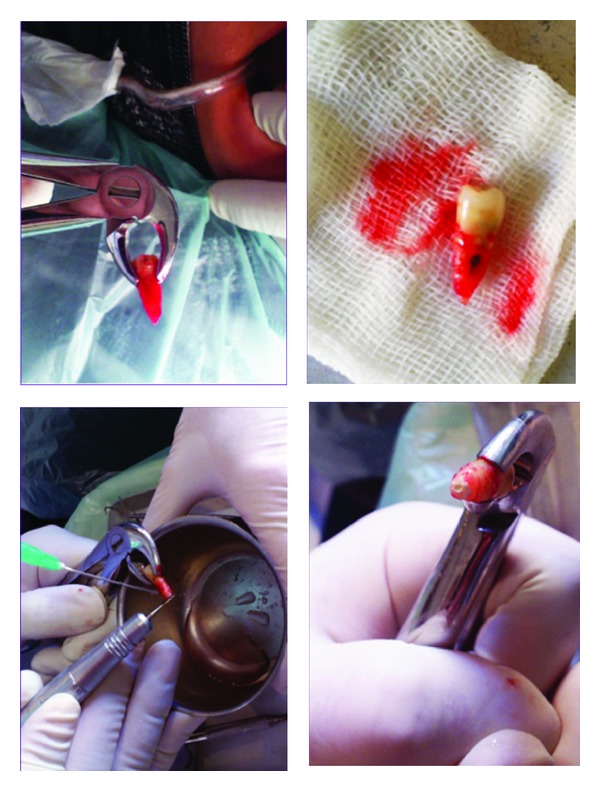
After the tooth extraction, apical 3 mm of the root apex was resected and the root end cavity was prepared and filled with CEM cement.

At the patient's return, antisepsis was carried out using 0.2% chlorhexidine gluconate; then, right mandibular second premolar was anesthetized using an IANB and long buccal nerve block injection (Lidocaine 2% with epinephrine 1 : 80000; Daroupakhsh, Tehran, Iran). The tooth was gently extracted by forceps with no intraoperative complications; subsequently, apical 3 mm of the root apex was resected and the root end cavity was prepared and filled with CEM cement (Figure 4).

**Figure 5:**
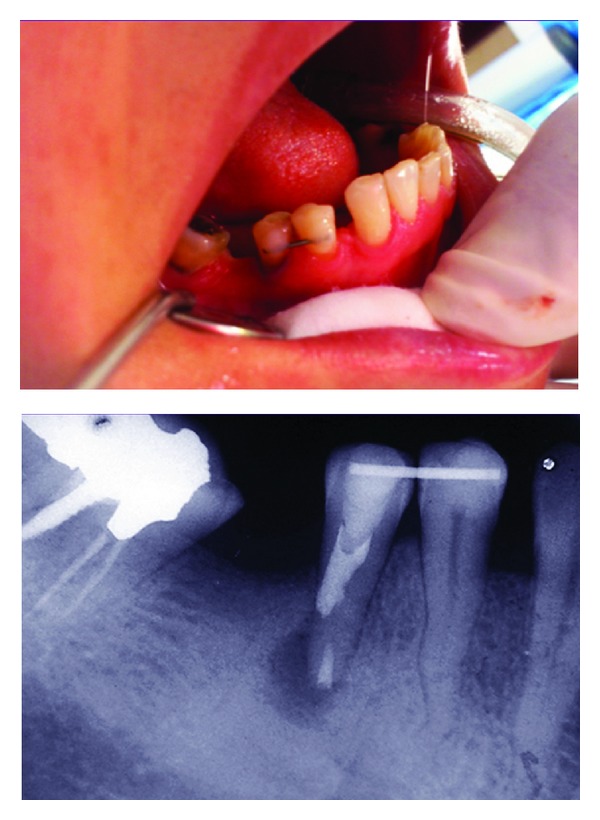
After replantation, the tooth was immobilized with a semirigid splint for 10 days.

Afterwards, the root surfaces were treated with tetracycline for 30 seconds to enhance the periodontal ligament cell attachment [7]. Next, the extracted tooth was replanted in its original position, and it was immobilized using a semirigid splint for 10 days (Figure 5). 4 × 400 mg ibuprofen, 0.2% chlorhexidine gluconate mouth rinse, and 3 × 500 mg amoxicillin daily for a week were prescribed.

## 3. Clinicoradiographical Followup

**Figure 6:**
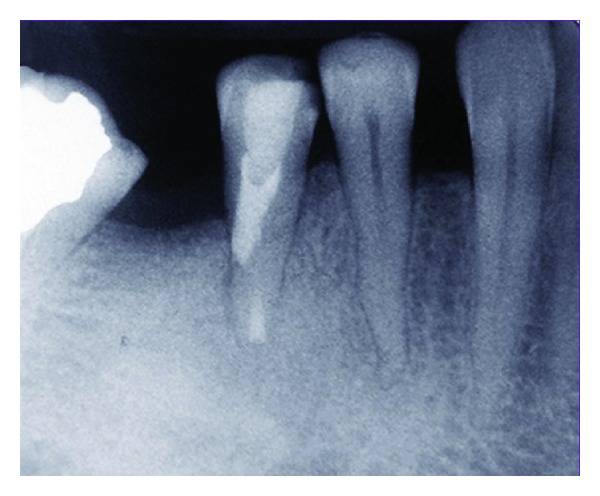
12 months after intentional replantation, periapical radiolucency was noticeably reduced.

Tooth's sensitivity to percussion and mobility were examined every three months. We evaluated the percussion tone and compared it with adjacent teeth. At 12 months after intentional replantation, no periodontal pocket was detected and the tooth was completely asymptomatic; it also had a slight degree of physiologic mobility. Furthermore, periapical radiolucency was noticeably reduced (Figure 6).

## 4. Discussion

When nonsurgical and surgical endodontic procedures have been deemed impossible and the patient desires all possible efforts be made to avoid tooth extraction and implant replacement, intentional replantation could be considered as the last treatment option [8].

Extraction and replantation of the tooth has been performed to manage several different problematic cases such as vertically fractured tooth [9], periodontally compromised hopeless tooth [10], calcified tooth [5], and iatrogenic perforation [11].

As described before, surgical endodontic treatment for mandibular premolars may lead to damaging adjacent vital structure such as the contents of mental foramen [12]; thus, before treatment planning, the risk for developing mental paresthesia after apicoectomy was seriously considered.

**Figure 7:**
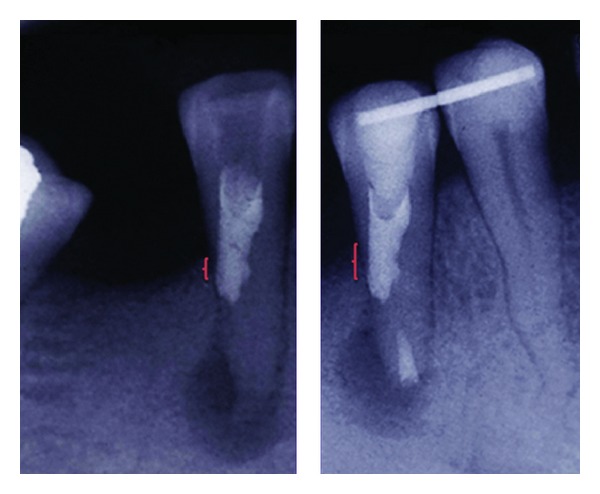
The distance between perforation site and the alveolar crest that has been shown with the red brackets was increased after root resection and tooth replantation.

In addition, there was an iatrogenic perforation on the distal root surface 1 mm below the alveolar crest. The short distance between the perforation area and the alveolar crest was a cause for concern, because there was danger of occurring bone loss and forming a periodontal pocket in the area [13], but after root resection and replantation the tooth was placed about 2 mm more apically than before; in so doing, the distance between the perforation site and the alveolar crest was increased (Figure 7).

In order to seal the apex of the tooth number 29, its root was resected and retrofilled with CEM cement. CEM cement is a biocompatible biomaterial [14] which is demonstrated that has an acceptable sealing ability when it is used to seal the root end cavities [15] and furcal perforations [16]. Also, it has been shown that in comparison to mineral trioxide aggregate (MTA), CEM cement's apical plug has superior sealing ability [17]. Therefore, in this case CEM cement was used to seal the perforation site and root end cavity.

The presence of healthy cementum on the root surface is one of the most important factors in prevention of ankylosis [18]. In order to produce a root surface that is conductive to cellular adhesion and growth, several solutions such as using tetracycline, citric acid, and ethylenediaminetetraacetic acid (EDTA) have been suggested [19]. In addition, in the previous studies [9, 20] tetracycline was used to treat the subjected teeth for thirty seconds just before the replantation. On the basis of these findings, in this case, tetracycline was applied to the root surfaces to enhance periodontal ligament fiber attachment and prevent ankylosis.

We evaluated the tooth's mobility and percussion sound during our controls to detect the ankylosis; because we know that the initial locations of ankylosis are usually on the lingual and/or labial tooth surfaces [21, 22], and it has been demonstrated that if an ankylotic area is located in these parts of a tooth, it will not be radiographically detectable [21].

After a year the right mandibular second premolar was mobile within normal limits, and the percussion tone was the same as that of the healthy adjacent tooth, but it is clear that monitoring this tooth for a long period of time is favorable.

## 5. Conclusion

Intentional replantation of the necrotic calcified teeth could be considered as an alternative to teeth extraction, especially for the single-rooted teeth and when nonsurgical and surgical endodontic procedures seem impossible.
